# Strict Time-Resolved Steady States via Affine-Eigenstate Mapping: A Robust Framework for Ultracold Atom–Molecule Dynamics

**DOI:** 10.3390/e28070752

**Published:** 2026-07-01

**Authors:** Yanhang Chen, Gaoyang Du, Chenglong Yang, Shuyu Dai, Bo Cui

**Affiliations:** 1 Leicester International Institute, Dalian University of Technology, Panjin 124221, China; 2549210234@mail.dlut.edu.cn (Y.C.); guishanshenqu@mail.dlut.edu.cn (G.D.); 2 CAS Key Laboratory of Mechanical Behaviour and Design of Materials, Department of Modern Mechanics, University of Science and Technology of China, Hefei 230027, China; chenglongyang@mail.ustc.edu.cn; 3 Key Laboratory of Materials Modification by Laser, Ion and Electron Beams (Ministry of Education), School of Physics, Dalian University of Technology, Dalian 116024, China; daishuyu@dlut.edu.cn; 4 School of General Education, Dalian University of Technology, Panjin 124221, China

**Keywords:** atom–molecule conversion, ultracold atom, polyatomic molecule, strict self-trapping, affine eigenstates

## Abstract

We propose a theoretical framework based on an affine-eigenstate transformation for analyzing ultracold atom–molecule conversion dynamics with particle loss. The transformation maps the mean-field dynamics to an effective two-mode representation in which fixed points, Bloch-sphere trajectories, and linear stability can be examined in a common set of variables. We give the derivation of the transformed Hamiltonian and specify the invertibility and conjugate-condition requirements under which the mapping is used. Within this representation, we distinguish ordinary, pseudo, and strict self-trapping regimes. The strict regime is associated with the balanced condition S=0 in the transformed variables; in the corresponding linearized dissipative flow, the leading attractor/repeller bifurcation term controlled by $S\Gamma_{-}$ vanishes, explaining the observed robustness against atom- and molecule-loss imbalance. We also introduce von Neumann and linear-entropy diagnostics for future mixed-state or ensemble descriptions in the transformed two-level representation, and we provide an inverse reconstruction procedure for preparing initial states that realize strict self-trapping. Finally, we discuss the limits of the mean-field and Markovian approximations and outline how finite-particle simulations and phase-modulated control protocols could connect this mechanism to decoherence-resilient quantum simulations and information-processing architectures.

## 1. Introduction

The coherent control of ultracold atom–molecule conversion systems has emerged as a cornerstone for quantum simulation and precision metrology. These systems inherently involve complex two-body interactions, where the interplay between quantum tunneling and nonlinear effects governs the population transfer between atomic and molecular modes. Although early studies relied on mean-field approximations (e.g., Gross–Pitaevskii equations) to reduce the dynamics to a two-mode model, such approaches often obscure the role of phase coherence and environmental dissipation—key factors in practical quantum control [1,2,3,4,5,6,7,8].

Recent advances in cavity and circuit quantum electrodynamics (QED) platforms have enabled the experimental exploration of analogous phenomena in photonic systems, such as self-trapping and multistability in coupled cavities. For example, seminal work by Albiez et al. demonstrated macroscopic self-trapping in Bose–Einstein condensates (BECs) trapped in double-well potentials, highlighting the critical role of nonlinearity in suppressing tunneling [9]. Subsequent studies extended this concept to dissipative systems, revealing that self-trapped states can exhibit finite lifetimes under photon loss or decoherence [6]. However, a unified framework to classify self-trapping regimes based on their robustness against environmental perturbations remains absent.

Dissipation and decoherence can qualitatively reshape nonlinear dynamics by changing the stability and even the nature of fixed points in phase space, as described within the standard Markovian master-equation framework [10,11,12]. In this work, we ask how particle losses with rates $\Gamma_{a}$ and $\Gamma_{b}$ reorganize the mean-field phase portrait of atom–molecule conversion, and whether a self-trapped state can remain robust rather than turning into an attractor/repeller-dominated metastable behavior under strong losses [13,14]. To address these questions, we introduce an affine-eigenstate transformation that maps the original variables onto a standard Bloch-sphere representation, which makes fixed points and their stability directly accessible. In the symmetric case R=0, we show that dissipative bifurcations admit a compact operational rule, which determines whether the relevant fixed point becomes attractive or repulsive, thus explaining the fragility of ordinary self-trapping. Most importantly, we identify a strict self-trapping regime associated with S=0, for which this bifurcation mechanism becomes inactive and the localization remains unusually robust against decoherence.

This question has become more timely because recent work has rapidly expanded both the experimental and theoretical basis for controllable atom–molecule and molecular many-body dynamics. New experiments and analyses have reported ultracold field-linked tetratomic molecules, molecular Bose condensation, narrow-resonance coherent atom–molecule oscillations, and Bose-enhanced relaxation in driven atom–molecule condensates [15,16,17,18]. Parallel developments in ultracold molecular arrays, polar-molecule quantum gases, self-bound molecular droplets, and ultracold mixtures highlight the relevance of long-lived internal states, tunable Feshbach coupling, and dipolar interactions for quantum simulation and information processing [19,20,21,22,23]. Recent studies of the atom–molecule phase structure also emphasize that coherent three-body recombination, metastability, and atom–molecule entanglement can strongly modify the effective two-mode picture [24]. These advances motivate the present focus on a transparent stability criterion and on explicit statements of the assumptions under which the affine mapping remains valid.

This paper is organized as follows: In Section 2, we derive the effective Hamiltonian and symmetry constraints using affine-eigenstate transformations. Section 3 and Section 4 analyze phase-space dynamics and self-trapping classification. Section 5 discusses the impact of time-varying magnetic fields and experimental implications for quantum state control in cavity QED setups, while Section 6 concludes with future directions.

## 2. Model

For an atomic–polyatomic molecule conversion system, the Hamiltonian can be expressed in the following form by using the Gross–Pitaevskii equations within the mean-field theory:

(1) $$\begin{array}{cc} H= & \mu_{a}{\hat{a}}^{†}\hat{a}+\mu_{b}{\hat{b}}^{†}\hat{b}+U_{aa}{\hat{a}}^{†}{\hat{a}}^{†}\hat{a}\hat{a}+U_{bb}{\hat{b}}^{†}{\hat{b}}^{†}\hat{b}\hat{b}\\ \; & +U_{ab}{\hat{a}}^{†}\hat{a}{\hat{b}}^{†}\hat{b}+V\;\left[{\left({\hat{a}}^{†}\right)}^{N}\hat{b}+{\hat{b}}^{†}{\left(\hat{a}\right)}^{N}\right]. \end{array}$$

In this form, the complex two-body problem is simplified to a two-mode system, which includes the following dimensionless parameters: atom–atom collision $U_{aa}$, molecule–molecule coupling $U_{bb}$, atom–molecule coupling $U_{ab}$, and atom–molecule conversion with rate *V*. *N* denotes the number of atoms that make up a molecule.

$l_{diss}\left(\rho \right)$ is the Liouville operator [25,26,27] containing creation and annihilation operators of atoms and molecules (${\hat{a}}^{†},{\hat{b}}^{†},\hat{a},\hat{b}$), representing the dissipation term in the master equation. The Lindblad master equation considering particle loss can be written as:

(2) $$\begin{array}{cc} l_{\mathrm{diss}}\left(\rho \right)= & \frac{\Gamma_{a}}{2}\left(2\hat{a}\rho {\hat{a}}^{†}-{\hat{a}}^{†}\hat{a}\rho -\rho {\hat{a}}^{†}\hat{a}\right)\\ \; & +\frac{\Gamma_{b}}{2}\left(2\hat{b}\rho {\hat{b}}^{†}-{\hat{b}}^{†}\hat{b}\rho -\rho {\hat{b}}^{†}\hat{b}\right), \end{array}$$

(3) $$\dot{\rho }=-i\left[\hat{H},\rho \right]+l_{\mathrm{diss}}\left(\rho \right).$$

where $\Gamma_{a}$ and $\Gamma_{b}$ are the single-particle loss rates of atoms and molecules, respectively. In the mean-field approximation, the quantum fluctuation is neglected and the operators $\hat{a}$ and $\hat{b}$ can be replaced by the *c* numbers $a=\left|a\right|e^{i\theta_{a}}$ and $b=\left|b\right|e^{i\theta_{b}}$ [3]. Taking these factors into account, and according to the trace calculation based on operators $\dot{\hat{a}}=Tr\left(\dot{\rho }\hat{a}\right)$, the master equation is reduced, under a mean-field (coherent-state) approximation, to the following Gross–Pitaevskii-type nonlinear equations: The reduction can be made explicit by evaluating $d\langle \hat{a}\rangle /dt=\mathrm{Tr}\left(\dot{\rho }\hat{a}\right)$ and $d\langle \hat{b}\rangle /dt=\mathrm{Tr}\left(\dot{\rho }\hat{b}\right)$. The commutator with Equation (1) gives the coherent part, whereas the Lindblad terms in Equation (2) contribute the damping terms $-\Gamma_{a}a/2$ and $-\Gamma_{b}b/2$. After the coherent-state replacement, one obtains

(4) $$\begin{array}{cc} i\dot{a}= & \left(\mu_{a}+2U_{aa}{\left|a\right|}^{2}+U_{ab}{\left|b\right|}^{2}-\frac{i}{2}\Gamma_{a}\right)a+NV{\left(a^{*}\right)}^{N-1}b,\\ i\dot{b}= & \left(\mu_{b}+2U_{bb}{\left|b\right|}^{2}+U_{ab}{\left|a\right|}^{2}-\frac{i}{2}\Gamma_{b}\right)b+Va^{N}. \end{array}$$

Removing a common scalar energy shift, collecting the interaction terms in the atom-number-normalized population imbalance $s={\left|a\right|}^{2}-N{\left|b\right|}^{2}$, and using the definitions of *R* and *U* below converts Equation (4) into the compact two-component form

(5) $$i\;\frac{d}{dt}\left(\begin{array}{c} a\\ b \end{array}\right)=H_{t}\left(\begin{array}{c} a\\ b \end{array}\right),$$

(6) $$H_{t}=\left(\begin{array}{cc} R-Us-\frac{i}{2}\Gamma_{a} & NV{\left(a^{*}\right)}^{N-1}\\ Va^{N-1} & -NR+NUs-\frac{i}{2}\Gamma_{b} \end{array}\right).$$

$H_{t}$ represents the matrix expression of the effective two-mode mean-field Hamiltonian with respect to time t, where *R* is the energy difference between the two modes: $R=\frac{1}{2N}\left(N\mu_{a}-\mu_{b}+NU_{aa}-\frac{1}{N}U_{bb}\right)$, *U* is the coupling strength: $U=\frac{1}{2N}U_{ab}-\frac{1}{2}U_{aa}-\frac{1}{2N^{2}}U_{bb}$, and *s* is the population difference: $s={\left|a\right|}^{2}-N{\left|b\right|}^{2}$.

**Figure 1:**
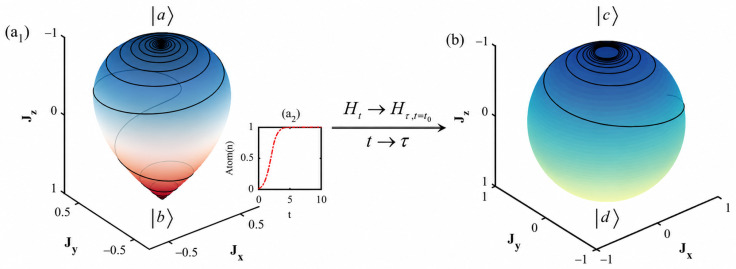
Affine-eigenstate transformation and time reparametrization. (**a_1_**) Original atom–molecule dynamics on a generalized Bloch surface with poles |a〉 and |b〉. (**a_2_**) Mapping from physical time *t* to effective time τ. (**b**) Transformed dynamics $\left(H_{t}\;\to \;H_{\tau },\;t\;\to \;\tau \right)$ on a standard Bloch sphere in the {|c〉,|d〉} basis.

By inserting the effective mean-field Hamiltonian into the nonlinear Schrödinger equation and subsequently resolving the differential equations through the fourth-order classical Runge–Kutta method, we can derive the evolution curves of the normalized total particle number $n_{nor}={\hat{a}}^{†}\hat{a}+N{\hat{b}}^{†}\hat{b}$ and Bloch vectors $\vec{h}=\left(J_{x},J_{y},J_{z}\right)$ for an atom–polyatomic molecule conversion system, as shown in Figure 1.

(7) h→t=2NRe(a*)Nb,2NIm(a*)Nb,N|b|2−|a|2.

Here, we contemplate a closed system $\left(\Gamma_{a}=\Gamma_{b}=0\right)$ while simultaneously neglecting the nonlinear terms $\left(U=0\right)$. The instantaneous eigenstates $|E_{\pm }\rangle$ and corresponding eigenenergy $E_{\pm }=-\frac{N-1}{2}R\pm \frac{1}{2}\sqrt{{\left(N+1\right)}^{2}R^{2}+4NV^{2}{\left|a_{0}\right|}^{2\left(N-1\right)}}$ of the atom–polyatomic molecule conversion system Hamiltonian under the initial state $\left(a=a_{0}\right)$ can be derived:

(8) $$|E_{+}\rangle =\left(\begin{array}{c} -NV{\left|a_{0}\right|}^{\;N-1}e^{-\left(N-1\right)i\theta_{a}}\\ \frac{N+1}{2}R-\frac{1}{2}\sqrt{{\left(N+1\right)}^{2}R^{2}+4NV^{2}{\left|a_{0}\right|}^{2(N-1)}} \end{array}\right),$$

(9) $$|E_{-}\rangle =\left(\begin{array}{c} -NV{\left|a_{0}\right|}^{\;N-1}\\ \left(\frac{N+1}{2}R+\frac{1}{2}\sqrt{{\left(N+1\right)}^{2}R^{2}+4NV^{2}{\left|a_{0}\right|}^{2(N-1)}}\right)e^{\left(N-1\right)i\theta_{a}} \end{array}\right),$$

$|E_{\pm }\rangle$ are in the space spanned by bases $|L\rangle$ and $|R\rangle$. Following a representation transformation, wherein the original time-independent bases $|L\rangle$ and $|R\rangle$ are replaced with $|E_{+}\rangle$ and $|E_{-}\rangle$, a revised set of equations is acquired.

In order to avoid ambiguity arising from the misuse of symbols, *t* is used to represent the parameter of the instantaneous eigenvector $|E_{\pm }\rangle$, $\theta_{a}=\omega_{a}t$. τ is employed to denote the time evolution under the action of $H_{\tau }$. It is noteworthy that the state evolves not with respect to *t* under the influence of Hamiltonian, but with respect to τ. The nonlinear Schrödinger equation for Equation (3) is as follows:

(10) $$i\frac{d}{d\tau }\left(\begin{array}{c} c(\tau )\\ d(\tau ) \end{array}\right)=H_{\tau ,t=t_{0}}\;\left(\begin{array}{c} c(\tau )\\ d(\tau ) \end{array}\right),$$

which exists as a conversion relation between states: For compactness, define the conversion coefficient in the eigenvector component as $A_{N}=NV{\left|a_{0}\right|}^{N-1}$. The inverse projection from the instantaneous-eigenstate amplitudes to the original amplitudes is then

(11) $$a\left(\tau \left(t\right)\right)=\langle L\;;\;0|\psi \;;\;t\rangle =-A_{N}\;c\left(t\right)\;e^{-\left(N-1\right)i\theta_{a}}-A_{N}\;d\left(t\right),$$

(12) $$b\left(\tau \left(t\right)\right)=\langle R\;;\;0|\psi \;;\;t\rangle =E_{R+}\;c\left(t\right)+E_{R-}\;d\left(t\right)\;e^{\left(N-1\right)i\theta_{a}}.$$

Equations (11) and (12) define the transformation used throughout the paper. It is useful to write them as

(13) $$\left(\begin{array}{c} a\\ b \end{array}\right)=M\left(t\right)\left(\begin{array}{c} c\\ d \end{array}\right),\;M\left(t\right)=\left(\begin{array}{cc} -A_{N}e^{-\left(N-1\right)i\theta_{a}} & -A_{N}\\ E_{R+} & E_{R-}e^{\left(N-1\right)i\theta_{a}} \end{array}\right).$$

The mapping is one-to-one whenever

(14) $$\det M\left(t\right)=-A_{N}\left[E_{R-}-E_{R+}\right]\neq 0.$$

This excludes the trivial uncoupled limits V=0 or $|a_{0}|=0$ and the degeneracy $E_{R-}=E_{R+}$. For the parameter windows used in the numerical figures, Equation (14) remains nonzero, so the inverse state reconstruction is well defined. The phase factors cancel in the determinant of this two-column mapping, so there is no additional phase-dependent singularity in Equation (14). Under this condition, the inverse relation is

(15) $$\left(\begin{array}{c} c\\ d \end{array}\right)=M^{-1}\left(t\right)\left(\begin{array}{c} a\\ b \end{array}\right),\;M^{-1}\left(t\right)=\frac{1}{\det M(t)}\left(\begin{array}{cc} E_{R-}e^{\left(N-1\right)i\theta_{a}} & A_{N}\\ -E_{R+} & -A_{N}e^{-\left(N-1\right)i\theta_{a}} \end{array}\right).$$

The later Bloch-sphere description therefore does not claim a new Hilbert space; it is a change in coordinates for the same two-amplitude mean-field state, followed by normalization in the transformed amplitudes.

To simplify the expression of $H_{\tau }$ in Equation (10), the following notations are introduced: the rescaled conversion rate $\upsilon_{0}=V{\left({a_{0}}^{*}\right)}^{N-1}\cdot V{\left(a_{0}\right)}^{N-1}=V^{2}{\left|a_{0}\right|}^{2(N-1)}$ and non-linearity $\delta_{1}=R\left(R-Us\right)$, $\delta_{2}=Us$. The initial population difference *s* is composed of three nonlinear terms: $s_{1}={\left|c\right|}^{2}-{\left|d\right|}^{2}$, $s_{2}={\left|c\right|}^{2}+{\left|d\right|}^{2}$, and $\zeta =c^{*}d\cdot e^{\left(N-1\right)i\theta_{a}}+cd^{*}\cdot e^{-\left(N-1\right)i\theta_{a}}$; the expression for *s* is given by:

(16) $$\begin{array}{c} s=\frac{N(N+1)}{2}R\sqrt{{\left(N+1\right)}^{2}R^{2}+4N\nu_{0}}\cdot s_{1}\\ \;-\frac{N{\left(N+1\right)}^{2}}{2}R^{2}\cdot s_{2}+2N^{2}\nu_{0}\cdot \zeta \end{array}$$

$s_{2}=1$ is the new normalization condition in $H_{\tau }$. $s_{1}$ and ζ are the population imbalance and phase coherence [28] between the atom and molecule modes, respectively. The intermediate expansion leading to Equation (16) is given in Appendix A; it follows directly from substituting the affine mapping into $s={\left|a\right|}^{2}-N{\left|b\right|}^{2}$.

Other important physical quantities are:

(17) $$E_{R+}=\left|\langle R|E_{+}\rangle \right|=\frac{N+1}{2}R-\frac{1}{2}\sqrt{{\left(N+1\right)}^{2}R^{2}+4N\nu_{0}},$$

(18) $$E_{R-}=\left|\langle R|E_{-}\rangle \right|=\frac{N+1}{2}R+\frac{1}{2}\sqrt{{\left(N+1\right)}^{2}R^{2}+4N\nu_{0}},$$

(19) $$E_{++}=-i\langle E_{+}|{\dot{E}}_{+}\rangle =-N^{2}\left(N-1\right)\omega_{a}\nu_{0},$$

(20) $$E_{+-}=-i\langle E_{+}|{\dot{E}}_{-}\rangle =-N\left(N-1\right)\omega_{a}\nu_{0}\;e^{\left(N-1\right)i\theta_{a}},$$

(21) $$E_{-+}=-i\langle E_{-}|{\dot{E}}_{+}\rangle =-N^{2}\left(N-1\right)\omega_{a}\nu_{0}\;e^{-\left(N-1\right)i\theta_{a}},$$

(22) $$E_{--}=-i\langle E_{-}|{\dot{E}}_{-}\rangle =\left(N-1\right)\omega_{a}{\left[\frac{N+1}{2}R+\frac{1}{2}\sqrt{{\left(N+1\right)}^{2}R^{2}+4N\nu_{0}}\right]}^{2},$$

$E_{R\pm }$ depends on the instantaneous eigenstates under the initial state of the conversion system. $E_{-+}=N{\left(E_{+-}\right)}^{*}$ is defined as the tunneling-induced factor. $E_{++}$ and $E_{--}$ are Berry connections [28], which are strongly correlated with the geometric phase factor and the quantum adiabatic evolution of the system; this will be discussed extensively in Section 3.

Based on the aforementioned notation settings and Equations (3) and (6), and considering the decoherence effects caused by the external environment, which can be treated as a Markovian process, the Hamiltonian in Equation (10) can be written as follows:

Starting from $\psi_{LR}=M\left(t\right)\psi_{cd}$ with $\psi_{LR}={\left(a,b\right)}^{T}$ and $\psi_{cd}={\left(c,d\right)}^{T}$, the transformed equation is obtained as

(23) $$i\frac{d\psi_{cd}}{d\tau }=\left[M^{-1}H_{t}M-iM^{-1}\frac{dM}{d\tau }\right]\psi_{cd}\equiv H_{\tau }\psi_{cd}.$$

The first term transports the nonlinear mean-field Hamiltonian to the instantaneous-eigenstate basis, and the second term gives the diagonal and off-diagonal geometric couplings listed in Equations (19)–(22). Substituting the coefficients in Equations (17)–(22) and the definitions of $\delta_{1}$, $\delta_{2}$, and $\nu_{0}$ yields the matrix elements below. The dissipative rates are not transformed into new phenomenological constants; in this mean-field Lindblad reduction, they enter as the same single-particle loss rates on the corresponding amplitude equations.

(24) $$H_{\tau ,t=t_{0}}=\left(\begin{array}{cc} H_{\tau (1,1)}-\frac{i}{2}\Gamma_{a} & H_{\tau (1,2)}\\ H_{\tau (2,1)} & H_{\tau (2,2)}-\frac{i}{2}\Gamma_{b} \end{array}\right),$$

(25) $$\left\{\begin{array}{c} H_{\tau (1,1)}=-N\left(N+1\right)\left(\delta_{1}+\nu_{0}\right)E_{R+}+E_{++},\\ H_{\tau (1,2)}=\left[N\left(N-1\right)E_{-}-2N^{2}\delta_{2}\right]\nu_{0}\;e^{\left(N-1\right)i\theta_{a}}+E_{+-},\\ H_{\tau (2,1)}=\left[N\left(N-1\right)E_{+}-2N^{2}\delta_{2}\right]\nu_{0}\;e^{-\left(N-1\right)i\theta_{a}}+E_{-+},\\ H_{\tau (2,2)}=-N\left(N+1\right)\left(\delta_{1}+\nu_{0}\right)E_{R-}+E_{--} \end{array}\right.$$

The probability amplitude is denoted by $c=\left|c\right|e^{i\theta_{c}}$ and $d=\left|d\right|e^{i\theta_{d}}$, the population imbalance $Z=s_{1}={\left|c\right|}^{2}-{\left|d\right|}^{2}$, and the relative phase $\Theta =\theta_{c}-\theta_{d}$.

Unlike previous studies, our approach involves using the instantaneous eigenstates of the Hamiltonian (IEHs) as the bases, resulting in the time-dependent evolution of the system. By using a rotating magnetic field with a frequency ω, we can effectively drive this two-level system. *Pseudo fixed points* are defined [29] by

(26) $$\dot{Z}=0\;\mathrm{and}\;\dot{\Theta }=\omega ,$$

for which the relative phase Θ=ωτ.

Analogous to the classical atom–polyatomic molecule conversion system, we conducted a dynamical analysis of the system after changing the basis vectors, and observed significant differences in the dynamical properties before and after the change. The most noticeable change was the quadratic coefficient of the population imbalance *Z* becoming ±1, resulting in the Bloch vectors spanning a Hilbert space that transforms from a tear-shaped structure to a classical spherical shape. The completeness of the Hilbert space allows the state vector $|\psi \rangle =\left|c\right||E_{+}\rangle +\left|d\right|e^{-i\Theta }|E_{-}\rangle$ of the system at any moment to be represented by a point on the surface of Bloch sphere, as depicted in Figure 1 [30,31,32,33].

(27) $${\vec{h}}_{\tau }=\left\{\sin\;(\arccos\;(Z))\cdot \cos\;(\Theta ),\;\sin\;(\arccos\;(Z))\cdot \sin\;(\Theta ),\;Z\right\}.$$

## 3. Symmetric Double-Well Case: R = 0

### 3.1. Effective Classical Hamiltonian and Phase-Space Structure

Based on the two-mode approximation of the nonlinear Schrödinger equation, we approximated the particles in the ultracold atom–polyatomic molecule conversion system as a Bose–Einstein condensate (BEC) trapped in a double-well potential. When setting R=0, the oscillations between atomic and molecular modes can be interpreted as quantum tunneling behavior occurring between two symmetric double wells.

Under this circumstance, the parameters in Equations (16) and (25) are simplified to:

(28) $$i\;\frac{d}{d\tau^{\prime }}\left(\begin{array}{c} c(\tau^{\prime })\\ d(\tau^{\prime }) \end{array}\right)=H\left(\begin{array}{c} c(\tau^{\prime })\\ d(\tau^{\prime }) \end{array}\right),$$

(29) $$H=\left(\begin{array}{cc} h_{11} & h_{12}\\ h_{21} & h_{22} \end{array}\right),\;\left\{\begin{array}{c} h_{11}=N\left[\left(N+1\right)\sqrt{N\nu_{0}}-N\left(N-1\right)\omega_{a}\right]\nu_{0},\\ h_{12}=N\left(N-1\right)\left(-\sqrt{N\nu_{0}}-\frac{2N}{N-1}\delta_{2}-\omega_{a}\right)\nu_{0}\;e^{\left(N-1\right)i\theta_{a}},\\ h_{21}=N\left(N-1\right)\left(\sqrt{N\nu_{0}}-\frac{2N}{N-1}\delta_{2}-N\omega_{a}\right)\nu_{0}\;e^{-\left(N-1\right)i\theta_{a}},\\ h_{22}=N\left[-\left(N+1\right)\sqrt{N\nu_{0}}+\left(N-1\right)\omega_{a}\right]\nu_{0}. \end{array}\right.$$

By abbreviating the two bases associated with τ as *c* and *d*, the nonlinear Schrödinger equation in this scenario is expressed as:

(30) $$z=2N^{2}V^{2}\;{\left|a\right|}^{2(N-1)}\left(c^{*}d\;e^{\left(N-1\right)i\theta_{a}}+cd^{*}\;e^{-\left(N-1\right)i\theta_{a}}\right),$$

(31) $$c=\left|c\right|\;e^{i\theta_{c}},$$

(32) $$d=\left|d\right|\;e^{i\theta_{d}}.$$

It is evident that, compared to the original form in Equation (3), the Hamiltonian of the atom–molecule conversion system undergoes a basis transformation, where the nonlinear term *U* on the main diagonal shifts to the off-diagonal, while the phase information from the initial state θ remains embedded in the off-diagonal elements of the transformed Hamiltonian. Our prior studies confirmed that nonlinearity can disrupt the adiabaticity of atomic BEC double-well systems, thereby modifying inter-well tunneling dynamics [34]. We thus inferred that the positional redistribution of these physical terms may trigger additional nontrivial phenomena.

Fixed points are critical factors governing the evolution trajectories of closed quantum systems in Hilbert space, while the phase space of the classical Hamiltonian effectively characterizes the variations in these fixed points [29,35,36]. To decode the phase-space information, we redefine the population imbalance *s* and phase difference θ for the basis-transformed system:

(33) $$\dot{s}=-4N^{4}u\nu_{0}^{2}\left(1-s^{2}\right)\sin2\theta -N\left(N-1\right)\left(N+1\right)\omega_{a}\nu_{0}\sqrt{1-s^{2}}\;\sin\theta ,$$

(34) $$\begin{array}{c} \dot{\theta }=\left[N\left(N-1\right)\left(N+1\right)\omega_{a}-2N\left(N+1\right)\sqrt{N\nu_{0}}\right]\nu_{0}\\ \;+N\left(N-1\right)\left[-\frac{8N^{3}}{N-1}u\nu_{0}s\cos\theta -\left(N+1\right)\frac{\omega_{a}s}{\sqrt{1-s^{2}}}+\frac{2\sqrt{N\nu_{0}}-\left(N-1\right)\omega_{a}}{\sqrt{1-s^{2}}}\right]\nu_{0}\cos\theta . \end{array}$$

The variables $\dot{s}$ and $\dot{\theta }$ form a pair of conjugate variables with respect to the classical Hamiltonian and satisfy $\omega_{a}=\frac{2\sqrt{N\nu_{0}}}{N-1}$.

Unlike conventional atomic BEC double-well systems, atom–polyatomic molecule conversion is inherently a many-body problem. We simplified it into a two-mode system (atomic and molecular modes) using the Gross–Pitaevskii (GP) equation under the mean-field approximation. However, in this approximation, the population imbalance *s* and phase difference θ are not fully canonically conjugate. To ensure the existence of the classical Hamiltonian, the approximation-induced deviations must be eliminated, which enforces the validity of the expression:

(35) $$\omega_{a}=\frac{2\sqrt{N\nu_{0}}}{N-1}=\frac{2\sqrt{N}}{N-1}\;V{\left|a\right|}^{N-1}.$$

We define this constraint as the conjugate condition. This condition plays a critical role in subsequent analyses, and its physical implications are rigorously addressed in Section 3.2. Mathematically, Equation (35) is the integrability condition for the reduced two-dimensional flow; it ensures that $\partial \dot{s}/\partial s+\partial \dot{\theta }/\partial \theta =0$ in the closed transformed dynamics and therefore that a scalar Hamiltonian $H_{e}\left(s,\theta \right)$ exists with $\dot{s}=-\partial H_{e}/\partial \theta$ and $\dot{\theta }=\partial H_{e}/\partial s$. If Equation (35) is violated, (s,θ) can still be propagated numerically, but the closed-flow interpretation as canonical Hamiltonian motion is no longer valid. This is why the stability classification below is restricted to the parameter sets satisfying the conjugate condition.

Building on this framework, a closed atom–polyatomic molecule conversion system can be described by the following classical Hamiltonian using instantaneous eigenstates as the basis:

(36) $$\begin{array}{c} H_{e}\left(s,\theta \right)=2N^{4}u\nu_{0}^{2}\cos2\theta -4N^{4}u\nu_{0}^{2}s^{2}\cos^{2}\theta \\ \;+\left[N\left(N-1\right)\left(N+1\right)\omega_{a}-2N\left(N+1\right)\sqrt{N\nu_{0}}\right]\nu_{0}\;s+N\left(N-1\right)\left(N+1\right)\omega_{a}\nu_{0}\sqrt{1-s^{2}}\cos\theta \end{array}$$

**Figure 2:**
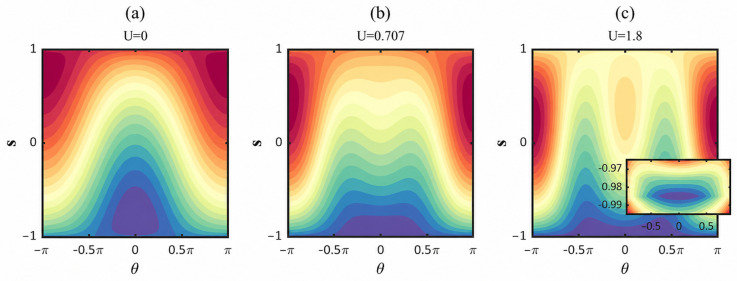
Phase-space trajectories for different nonlinear terms. The phase difference θ is measured in units of π (initial parameters). Nonlinear strengths are fixed at U=0 in (**a**), U=0.707 in (**b**), and U=1.8 in (**c**). For weak nonlinearity, two fixed points exist [panels (**a**,**b**)]. Beyond a critical value, two additional fixed points emerge in the phase space [panel (**c**)]. When the nonlinearity becomes sufficiently strong, the system becomes trapped near fixed polar regions. All parameters in this work are rescaled to dimensionless units.

The dependence of the classical Hamiltonian on the population imbalance *s* and phase difference θ is illustrated in Figure 2. Notably, the phase-space trajectories of the basis-transformed system exhibit a closer resemblance to those of the atomic BEC double-well model compared to the original system.

From a qualitative perspective, as the nonlinearity *U* increases beyond a critical threshold, new fixed points emerge in the phase space, while preexisting fixed points persist, but become progressively compressed toward the lower region of the phase space. Quantitatively, the positions and stability types of these fixed points constitute central observables.

To determine the fixed-point coordinates, we employed the defining conditions for pseudo fixed points: $\dot{s}=0$, $\dot{\theta }=0$, which yield the governing equations:

(37) $$\frac{\left[2\sqrt{N\nu_{0}}-\left(N-1\right)\omega_{a}\right]{\left(N-1\right)}^{2}\left(N+1\right)\omega_{a}}{8N^{2}u\left(1-s^{2}\right)}=\left[N\left(N-1\right)\left(N+1\right)\omega_{a}-2N\left(N+1\right)\sqrt{N\nu_{0}}\right]\nu_{0}.$$

A set of numerical solutions can readily be obtained: s=±1, $\omega_{a}=\frac{2\sqrt{N\nu_{0}}}{N-1}$

These solutions precisely satisfy the conjugate condition mentioned above. Similarly, the stability types of the fixed points can be resolved numerically.

The Jacobian matrix was introduced:

(38) $$J=\left(\begin{array}{cc} \frac{\partial \dot{s}}{\partial s} & \frac{\partial \dot{s}}{\partial \theta }\\ \frac{\partial \dot{\theta }}{\partial s} & \frac{\partial \dot{\theta }}{\partial \theta } \end{array}\right)$$

The individual derivatives used in the numerical stability map are

(39) $$\begin{array}{cc} J_{11}= & 8N^{4}U\nu_{0}^{2}s\sin2\theta +N\left(N-1\right)\left(N+1\right)\omega_{a}\nu_{0}\frac{s}{\sqrt{1-s^{2}}}\sin\theta ,\\ J_{12}= & -8N^{4}U\nu_{0}^{2}\left(1-s^{2}\right)\cos2\theta -N\left(N-1\right)\left(N+1\right)\omega_{a}\nu_{0}\sqrt{1-s^{2}}\cos\theta ,\\ J_{21}= & N\left(N-1\right)\nu_{0}\left[-\frac{8N^{3}}{N-1}u\cos\theta -\frac{\left(N+1\right)\omega_{a}}{{\left(1-s^{2}\right)}^{3/2}}+\frac{\left[2\sqrt{N\nu_{0}}-\left(N-1\right)\omega_{a}\right]s}{{\left(1-s^{2}\right)}^{3/2}}\right]\cos\theta ,\\ J_{22}= & N\left(N-1\right)\nu_{0}\left[\frac{8N^{3}}{N-1}us\sin\theta +\left(\frac{\left(N+1\right)\omega_{a}s}{\sqrt{1-s^{2}}}-\frac{2\sqrt{N\nu_{0}}-\left(N-1\right)\omega_{a}}{\sqrt{1-s^{2}}}\right)\sin\theta \right]. \end{array}$$

At each fixed point, Equation (39) is evaluated after enforcing Equations (33) and (34). The sign of $J_{12}J_{21}$ then separates center-type points from saddle-type points in the closed system, while the trace term determines the leading contraction or expansion once loss is introduced.

The stability of a system can be determined by calculating the eigenvalues of the state matrix. Under the standard methodology, the eigenvalues of the Jacobian matrix must be rigorously computed to classify the stability types—unstable, oscillatory stable, or asymptotically stable—across the parameter space [37,38]. For the classical Hamiltonian in Equation (36), the eigenvalues of the system’s Jacobian matrix are:

(40) $$\lambda_{\pm }=\frac{J_{11}+J_{22}}{2}\pm \frac{1}{2}\sqrt{{\left(J_{11}-J_{22}\right)}^{2}+4J_{12}J_{21}}.$$

For the closed Hamiltonian flow satisfying the conjugate condition, the trace $J_{11}+J_{22}$ vanishes at the fixed points, and Equation (40) reduces to the familiar center/saddle classification. We therefore used Equation (40) throughout rather than the older shorthand expression, which is valid only under additional trace and diagonal-entry assumptions.

As demonstrated in Figure 2, the phase space of the classical Hamiltonian was explicitly mapped, and its local extrema—corresponding to stable points—can be intuitively identified. However, the specific stability types of these points cannot be directly discerned. Consequently, our objective shifted from determining whether the system is stable or unstable under given parameters to quantifying its stability margin, which rigorously characterizes the robustness of fixed points against parametric perturbations.

**Figure 3:**
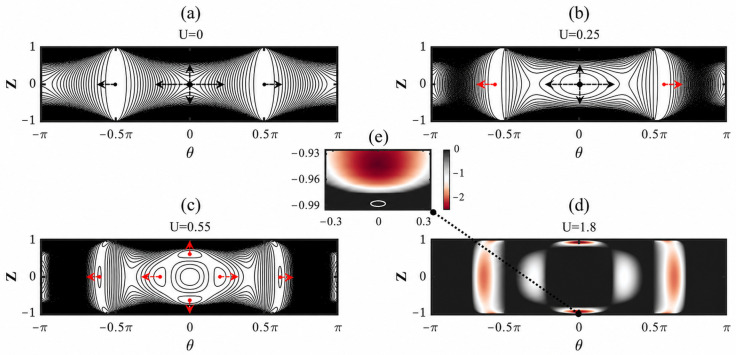
Stability of fixed points under varying nonlinear strengths.White regions indicate purely imaginary Jacobian eigenvalues (center-type, linearly stable), whereas colored regions correspond to real eigenvalues (saddle-type, linearly unstable). Nonlinear terms are fixed at U=0 in (**a**), U=0.25 in (**b**), U=0.55 in (**c**), and U=1.8 in (**d**,**e**). As the nonlinearity increases, additional elliptic fixed points emerge, consistent with the phase-space trajectories, while hyperbolic regions expand and shift in phase space. Concurrently, unstable hyperbolic fixed points expand radially and become progressively compressed near s=±1.

For the closed mean-field dynamics, the linear stability of a fixed point is determined by the eigenvalues of the Jacobian matrix. In a two-dimensional conservative flow, fixed points are naturally classified into center-type and saddle-type. If the Jacobian eigenvalues are purely imaginary, λ=±iω, small perturbations lead to bounded oscillations around the fixed point, which is therefore elliptic and linearly Lyapunov-stable. If the eigenvalues are real, λ=±κ, the fixed point is hyperbolic: perturbations grow exponentially along the unstable manifold, and the fixed point is linearly unstable. In a closed system, there are no attractors or repellers in the asymptotic sense. This asymptotic stability only emerges once dissipation is introduced. We therefore visualized the eigenvalue character over the entire phase space after the basis transformation, as shown in Figure 3.

As the nonlinearity *U* gradually increases and surpasses a critical threshold, new stable elliptical fixed points bifurcate in the phase space, while the preexisting hyperbolic fixed points are progressively compressed toward the lower region of the phase space.

### 3.2. Classification of Self-Trapping Regimes in Symmetric Systems

In this part, we focus on the self-trapping phenomena occurring in atomic–molecular two-mode energy-symmetric conversion systems. Based on factors such as the robustness against environmental perturbations, we identified three distinct forms of self-trapping phenomena in this context.

In the ultracold atom–molecule conversion process, the quantum system can be categorized into two modes: one involves oscillation and conversion between atomic and molecular modes, while the other exhibits the system being trapped in a single mode, known as self-trapping. Notably, both modes are subject to external environmental interference (dissipation and decoherence). Based on the master Equation (2), we continued to treat this interference as a unidirectional Markov process, neglecting the system’s back-action on the external environment.

**Figure 4:**
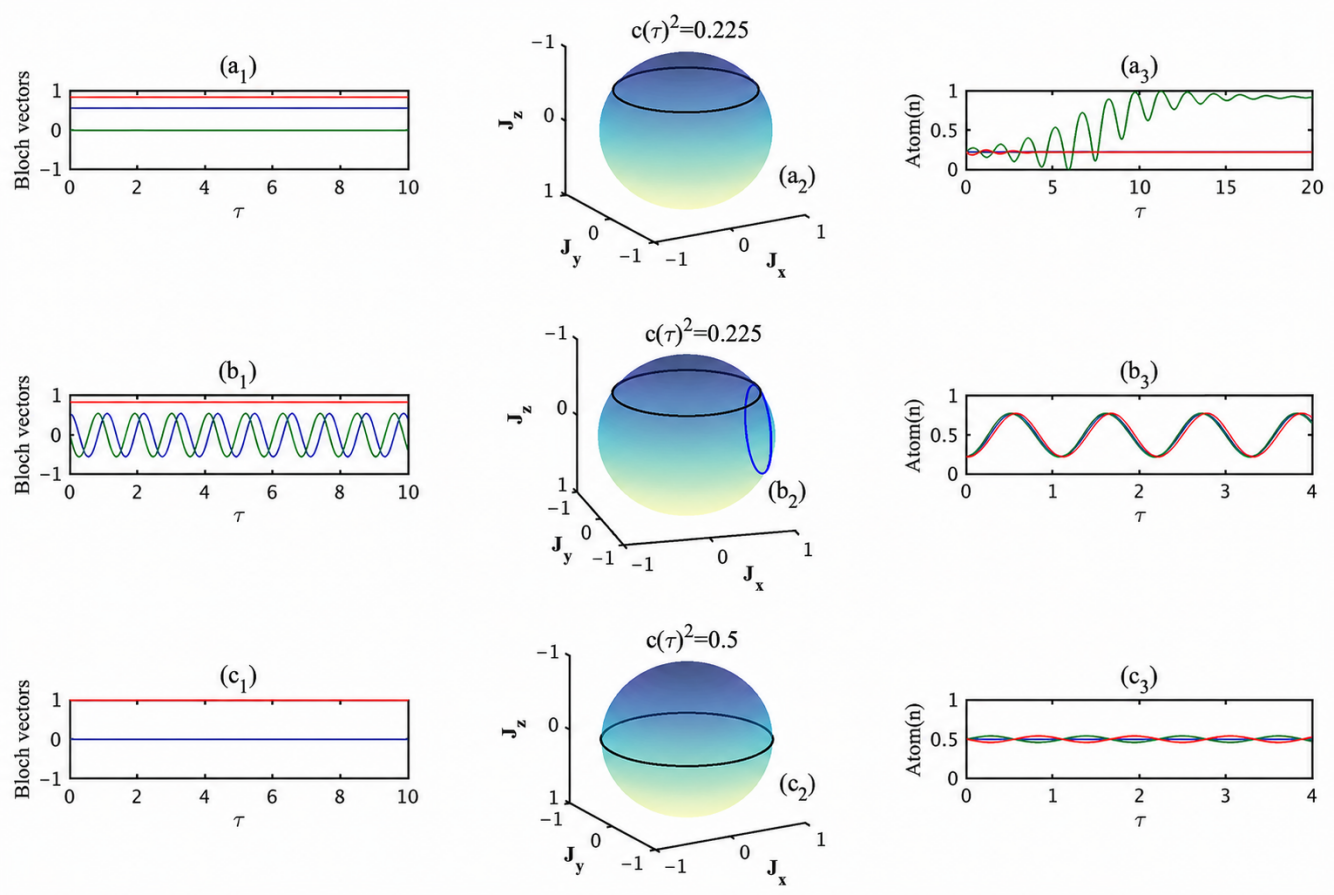
Three representative dynamical regimes of the transformed atom–molecule system. Rows (**a**–**c**) correspond to ordinary self-trapping, pseudo self-trapping, and strict self-trapping, respectively. The first column shows the evolution of the Bloch-vector components, the second column shows the corresponding Bloch-sphere trajectories, and the third column shows the atomic population evolution under dissipative conditions. In panel (**a_1_**–**c_1_**), the red, green, and blue curves represent $J_{x}$, $J_{y}$, and $J_{z}$, respectively.

By solving the nonlinear Schrödinger equation after the basis transformation, we obtained the Bloch vector characterizing the system’s evolutionary state. When the $J_{x},J_{y},J_{z}$ coordinates of the Bloch vector remain invariant with respect to τ, the population difference between atoms and molecules Δ maintains a constant value, indicating the occurrence of self-trapping, where the system persistently maintains its initial quantum state. In Hilbert space, this is manifested as a fixed point rather than a time-varying trajectory. Based on this foundation, we deliberately selected specific parameters, obtaining three sets of numerical solutions with distinct properties, as shown in Figure 4.

For two-mode energy symmetry, the most fundamental constraint condition R=0 must be satisfied. On this basis, when τ=0, we have $a\left(\tau \right)=|a_{0}|\cdot e^{\left(n-1\right)i\theta_{0}}$, meaning that, after the basis transformation, the system evolves with τ under the initial conditions of the original system, as shown in Figure 4a.

First, considering the closed system, under fixed values of the other parameters, by adjusting only the initial state c(τ) of the new system, there exists one and only one solution that leads the system to a self-trapping state. At this point, neither the population difference between the two modes nor the phase difference varies with τ, i.e., $\dot{Z}=0$, $\dot{\theta }=0$, satisfying the definition of fixed points in closed systems. Subsequently, considering the influence of the external environment, namely the decoherence effects in open quantum systems, after the basis transformation, the dissipative Hamiltonian takes the form:

(41) $$\begin{array}{c} H_{e}\left(S,\theta \right)=2N^{4}U\;\nu_{0}^{2}\cos2\theta -4N^{4}U\;\nu_{0}^{2}S^{2}\cos^{2}\theta \\ \;+\left(\left[N\left(N-1\right)\left(N+1\right)\omega_{a}-2N\left(N+1\right)\sqrt{N\nu_{0}}\right]\nu_{0}-\left(N-1\right)\omega_{a}\right)S\\ \;+N\left(N-1\right)\left(N+1\right)\omega_{a}\;\nu_{0}\sqrt{1-S^{2}}\cos\theta \end{array}$$

We discovered that, in open systems, the aforementioned self-trapping states are disrupted and show a particular sensitivity to molecular loss, as shown in Figure 4(a3). When the molecular loss rate $\Gamma_{b}$ exceeds the atomic loss rate $\Gamma_{a}$, the system gradually escapes from the self-trapping state through violent oscillations, and after a period of time, as the participating particles in the evolution are almost completely dissipated due to decoherence effects, the system approaches a new steady state. Conversely, when $\Gamma_{b}<\Gamma_{a}$, the system’s oscillations gradually weaken and eventually approach the self-trapping state of the closed system.

In previous work, we showed that fixed points in closed systems can bifurcate into attractors or repellers in open environments [39], and we revealed the mixing nature of this bifurcation [40,41]. In this paper, we identify the critical parameter values at which fixed points become attractors and those at which they become repellers. Again, the Jacobian matrix was introduced:

(42) $$\dot{S}=-2\sqrt{N}\;\sqrt{1-S^{2}}\;{\left(\frac{1+S}{2}\right)}^{\frac{N-1}{2}}\;\Omega \sin\theta -\Gamma_{-}\left(1-S^{2}\right),$$

(43) $$\dot{\theta }=\sqrt{N}\;\frac{\left(N+1)S-(N-1\right)}{\sqrt{1-S^{2}}}\;{\left(\frac{1+S}{2}\right)}^{\frac{N-1}{2}}\;\Omega \cos\theta -2N\left(R-CS\right).$$

Unlike Equations (26), here, we need to explore the stability of an open system (where repellers are unstable and attractors are stable). However, it is evident that, in the open quantum system after the basis transformation, deriving analytical expressions for population differences with decoherence terms Γ is extremely challenging. Therefore, we redirected our attention to the pre-transformation atom–multi-atom molecular conversion system, where $\dot{S}$, $\dot{\theta }$ respectively correspond to the rates of change in the population difference and phase difference with time *t* under particle loss consideration [39]. Furthermore, based on the definition of fixed points, the phase difference θ can be expressed in terms of other variables:

(44) $$\cos\theta =\frac{2N(R-CS)}{\sqrt{N}\cdot \frac{\left(N+1)S-(N-1\right)}{\sqrt{1-S^{2}}}\cdot {\left(\frac{1+S}{2}\right)}^{\frac{N-1}{2}}\cdot \Omega }$$

(45) $$\sin\theta =\frac{\tau_{-}\cdot \left(1-S^{2}\right)}{-2\sqrt{N}\cdot \sqrt{1-S^{2}}\cdot {\left(\frac{1+S}{2}\right)}^{\frac{N-1}{2}}\cdot \Omega }$$

Here, $\tau_{-}$ denotes the same relative loss parameter as $\Gamma_{-}$; in the following, we use $\Gamma_{-}$ consistently. The Jacobian of Equations (42) and (43) can be decomposed as

(46) $$J_{\mathrm{open}}=J_{\mathrm{coh}}+\left(\begin{array}{cc} 2\Gamma_{-}S & 0\\ 0 & 0 \end{array}\right),$$

where $J_{\mathrm{coh}}$ is the coherent part obtained at $\Gamma_{-}=0$ after substituting the fixed-point values of sinθ and cosθ. Thus, to the leading order near the self-trapped fixed point,

(47) $$\mathrm{Tr}\;J_{\mathrm{open}}=\mathrm{Tr}\;J_{\mathrm{coh}}+2\Gamma_{-}S.$$

For the closed transformed dynamics satisfying the conjugate condition, $\mathrm{Tr}\;J_{\mathrm{coh}}=0$. Hence, the real part of the linearized eigenvalues is controlled by $\Gamma_{-}S$ to the first order. Negative $\Gamma_{-}S$ gives local contraction and an attractor-like fixed point, while positive $\Gamma_{-}S$ gives local expansion and a repeller-like fixed point. The balanced strict case S=0 removes this leading dissipative contraction/expansion channel, which is the mathematical reason why it does not bifurcate in the same way as ordinary self-trapping.

If the real part of the eigenvalues in the Jacobian matrix Equation (38) is negative, it indicates that the non-Hermitian quantum system is stable, meaning that the fixed points will bifurcate into attractors, causing the evolution trajectory to approach the whirlpool center, and after a considerable period, the system’s quantum state remains constant. We systematically examined the variables in Equations (42) and (43), excluding the energy difference *R*, the normalized coupling strength C=U·n, and the normalized conversion rate $\Omega =V\cdot {\sqrt{n}}^{N-1}$ (where $n={\left|a\right|}^{2}+N{\left|b\right|}^{2}$ represents the total particle number), and ultimately determined the factors affecting the bifurcation type of fixed points: the normalized population difference $S=\frac{s}{n}$ and the relative loss rate $\Gamma_{-}=\frac{\Gamma_{a}-\Gamma_{b}}{2}$. By solving the eigenvalues of the Jacobian matrix, we can obtain the analytical expression for the eigenvalues:

(48) $$\lambda =\frac{\left(J_{11}+J_{22}\right)\pm \sqrt{{\left(J_{11}-J_{22}\right)}^{2}+4J_{12}J_{21}}}{2}$$

**Figure 5:**
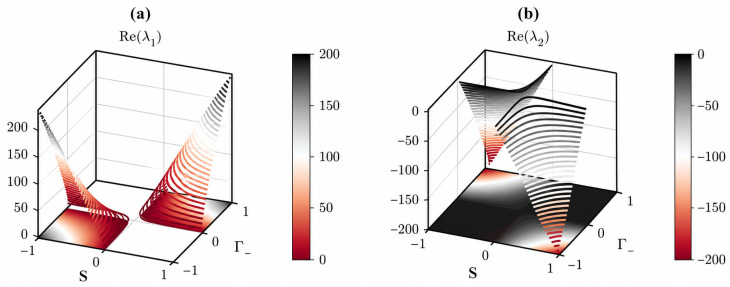
The sign relationship between the normalized population difference and the loss rate. Here, λ represents the eigenvalue. When their signs are the same, as shown in Figure (**a**), the eigenvalues lie in the first and third quadrants, and the fixed points bifurcate into repellers. Similarly, as shown in Figure (**b**), when their signs are opposite, the fixed points bifurcate into attractors.

When *S* and $\Gamma_{-}$ have opposite signs, the fixed points bifurcate into attractors; conversely, they bifurcate into repellers. The conclusion is shown in Figure 5.

Once dissipation is present, the mean-field flow is no longer governed by a conservative Hamiltonian structure. Instead, attractors and repellers may emerge and reorganize the phase portrait, so that long-time localization becomes a stability problem rather than a purely energetic one [42,43,44]. Linearizing the dissipative mean-field equations around a fixed point yields the Jacobian analysis summarized in Equations (42)–(48). In our parametrization, the resulting eigenvalue structure led to a particularly transparent rule: the dissipative bifurcation type is largely controlled by the sign relation between the normalized imbalance *S* and the relative loss rate $\Gamma_{-}=\left(\Gamma_{a}-\Gamma_{b}\right)/2$, such that the sign of $S\Gamma_{-}$ determines whether the fixed point behaves as a repeller or as an attractor. This sign rule provides a compact mechanism-level explanation for the numerical trends reported in Figure 4, and it is consistent with earlier phase-space analyses of loss-driven mean-field dynamics in related bosonic settings [42,43], as well as in atom–molecule conversion models with particle losses.

With this insight, we introduce a practical classification of self-trapping in the dissipative dynamics. We refer to the conventional self-trapped solutions—familiar from the closed-system nonlinear dynamics—as ordinary self-trapping. In a closed system, these solutions are governed by genuine fixed points and yield long-lived localization. Under dissipation, however, their stability depends sensitively on the sign of $S\Gamma_{-}$. When the relevant fixed point turns repulsive, trajectories are expelled from the self-trapped region and localization breaks down. Conversely, when it turns attractive, dissipation drives trajectories back toward the localized region and self-trapping is reinforced. In addition to this fixed-point-controlled behavior, we observed a decoherence-insensitive localized-like evolution that did not strictly satisfy the fixed-point definition of self-trapping. In this regime, time traces showed persistent localization with small-amplitude oscillations, while the phase portrait indicates that the trajectory was not organized by a true self-trapped fixed point; rather, it remained confined within a bounded region of phase space due to the local structure of the dissipative flow. We therefore termed this regime pseudo self-trapping, emphasizing that the apparent robustness did not originate from a genuine self-trapped equilibrium point.

Finally, the affine-eigenstate transformation allowed us to identify a special self-trapped state that is exceptionally robust against both atomic and molecular loss, which we call strict self-trapping. In the symmetric case R=0, strict self-trapping corresponds to a critically balanced configuration with a vanishing imbalance in the transformed representation. Because the dissipative bifurcation mechanism is controlled by $S\Gamma_{-}$, it becomes inactive at S=0: the fixed point does not split into an attractor/repeller pair and instead remains marginal in the linearized flow. This explains why strict self-trapping persists even for strong dissipation, displaying only minute residual oscillations rather than a qualitative breakdown. The three regimes may therefore be summarized operationally as follows: ordinary self-trapping is a fixed-point localization whose fate is controlled by the sign of $S\Gamma_{-}$; pseudo self-trapping is bounded localized motion without a true stationary point; and strict self-trapping is the balanced S=0 fixed-point case in which the leading dissipative bifurcation channel is absent. In the next subsection, we exploit this one-to-one correspondence between the steady solutions in the transformed picture and the initial conditions in the original variables to formulate an inverse reconstruction procedure that is useful for both stability diagnoses and state preparation.

### 3.3. Entropy and Mode-Correlation Diagnostics

To address whether the proposed steady regimes can be connected to atom–molecule correlation and entanglement, we added entropy diagnostics for a mixed or ensemble-averaged density matrix in the transformed two-mode representation. For a single deterministic mean-field trajectory, the normalized transformed amplitudes ${\tilde{\psi }}_{cd}={\left(\tilde{c},\tilde{d}\right)}^{T}$ define a rank-one density matrix

(49) $$\rho_{cd}=\frac{1}{{\left|c\right|}^{2}+{\left|d\right|}^{2}}\left(\begin{array}{cc} {\left|c\right|}^{2} & cd^{*}\\ c^{*}d & {\left|d\right|}^{2} \end{array}\right)=\frac{1}{2}\left(I+\vec{r}\cdot \vec{\sigma }\right),$$

where r→=(2Rec˜*d˜,2Imc˜*d˜,|c˜|2−|d˜|2) and $\vec{\sigma }$ are Pauli matrices. For a mixed state or ensemble-averaged state, the same Bloch representation has eigenvalues

(50) $$p_{\pm }=\frac{1\;\pm \;|\vec{r}|}{2},\;S_{\mathrm{vN}}=-\sum_{\alpha =\pm }p_{\alpha }\ln p_{\alpha },\;S_{L}=1-\mathrm{Tr}\rho_{cd}^{2}=\frac{1\;-\;|\vec{r}{|}^{2}}{2}.$$

For any single normalized mean-field trajectory, including a trajectory propagated with non-Hermitian loss and then renormalized, $\rho_{cd}$ remains at rank one, $|\vec{r}|=1$, and both $S_{\mathrm{vN}}$ and $S_{L}$ vanish. Nonzero values of Equations (49) and (50) therefore require a genuinely mixed density matrix, for example, one obtained by tracing over environmental degrees of freedom, averaging over stochastic quantum trajectories, or averaging over an ensemble of noisy initial conditions. In that mixed-state setting, $|\vec{r}|<1$ measures decoherence-induced mixing in the effective two-level description. The strict self-trapping condition S=0 corresponds to equal transformed populations, $|\tilde{c}{|}^{2}={\left|\tilde{d}\right|}^{2}$, so its robustness can be monitored by the persistence of $r_{z}=0$ together with a small ensemble or reduced-state entropy. A genuine atom–molecule entanglement entropy would require a many-body density matrix in the number basis, e.g., $\rho =\sum_{n,m;n^{\prime },m^{\prime }}\rho_{nm,n^{\prime }m^{\prime }}|n_{a},m_{b}\rangle \langle n_{a}^{\prime },m_{b}^{\prime }|$, followed by tracing over one mode. Such a calculation is outside the present mean-field approximation, but Equations (49) and (50) provide a feasible mixed-state diagnostic that can be compared with future quantum-trajectory or exact finite-number simulations.

### 3.4. Inverse Reconstruction of Initial States

In this part, we present a method for solving the initial state of the original system using the known steady state of the new system. First, we provide the definition of steady state in this context: from the moment t = 0 until the end of evolution, the system state remains unchanged and is almost unaffected by external influences, namely the strict self-trapping state in Section 3.2.

Based on the conclusions from the previous section, we know that the transformed system is in a strict self-trapping state when ${\left|c\left(\tau \right)\right|}^{2}=\frac{1}{2}$. Next, we performed an inverse transformation on the basis from Section 2; it can be readily shown that:

(51) $$\left|a(t)\right|=\left|-A_{N}e^{-\left(N-1\right)i\theta_{a}}\;c\left(\tau \right)-A_{N}\;d\left(\tau \right)\right|$$

(52) $$\begin{array}{c} \left|b\left(t\right)\right|=|\left[\frac{N+1}{2}R-\frac{1}{2}\sqrt{{\left(N+1\right)}^{2}R^{2}+4NV^{2}{\left|a_{0}\right|}^{2(N-1)}}\right]c\left(\tau \right)\\ \;+\left[\frac{N+1}{2}R+\frac{1}{2}\sqrt{{\left(N+1\right)}^{2}R^{2}+4NV^{2}{\left|a_{0}\right|}^{2(N-1)}}\right]e^{\left(N-1\right)i\theta_{a}}d\left(\tau \right)| \end{array}$$

Since a major prerequisite for strict self-trapping is R=0, c(t)=d(t), by substituting these conditions into Equations (51) and (52), we can obtain $\left|a(t)\right|=\sqrt{N}\left|b(t)\right|$.

That is, if the original system is an ultracold atom–multi-atom molecule conversion system, we can obtain the initial state of the original system under strict self-trapping conditions with a(t) and b(t) as the basis ${\left[\begin{array}{cc} \sqrt{\frac{N}{N+1}} & \sqrt{\frac{1}{N\left(N+1\right)}} \end{array}\right]}^{T}$.

This method provides a feasible and effective model for evaluating the stability of quantum systems. For an ultracold atom–multi-atom molecule conversion system, we can directly assess the stability under the initial conditions using this method, without the need for extensive and complex mathematical calculations. It is worth emphasizing that this method also has practical application value in fields such as quantum control, providing theoretical guidance for achieving atom–molecule conversion control in quasi-double-well systems.

### 3.5. Strict Self-Trapping in Droplet Phase Space

**Figure 6:**
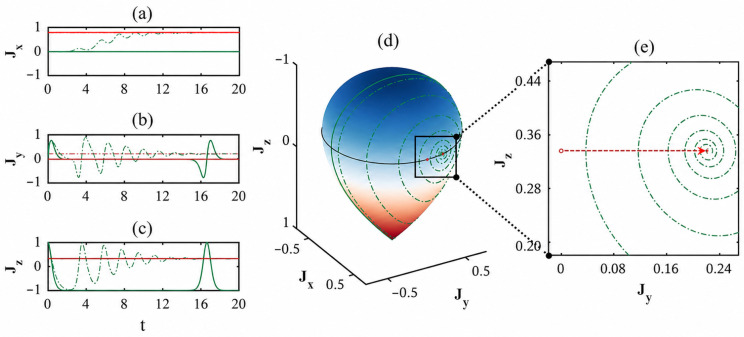
In Figures (**a**–**c**), green solid lines represent the evolution trajectories of arbitrary self-trapping points in closed systems, red solid lines represent the evolution trajectories of strict self-trapping points in closed systems, green dashed lines represent the evolution trajectories of arbitrary self-trapping points in open systems, and red dashed lines represent the evolution trajectories of strict self-trapping points in open systems. In Figure (**d**), the green solid line represents the evolution trajectory in the closed system, while the dashed line represents the evolution trajectory in the open system. In Figure (**e**), the solid red dot represents the strict self-trapping point (stable point), which slightly shifts by the ϕ phase when transitioning from a closed system to an open system.

After obtaining self-trapping cases with favorable properties in the transformed system, we naturally became curious about the form of these self-trapping points in the original system and how they change under environmental influences. By mapping the strict self-trapping points from the Bloch sphere back to the original droplet-shaped atom–molecule conversion system, and performing numerical simulations for both closed and open systems, we discovered that strict self-trapping points evolve only on the self-trapping plane, regardless of whether in closed or open systems, and exhibit a phase shift of ϕ as shown in Figure 6e. Meanwhile, ordinary self-trapping points bifurcate into either attractors or repellers during the transition from closed to open systems, displaying similar evolution trajectories. Based on these findings, we further confirmed the uniqueness and strong robustness of strict self-trapping.

It can be observed that the conclusions obtained in both the original and transformed systems are universal. This is because the one-to-one correspondence between the original system and the affine-eigenstate transformed system is a diffeomorphic mapping, similar to coordinate transformations in manifolds. We discuss these specific details in Section 4.

## 4. Asymmetric Double-Well Case: R≠0

In this section, we extend our conclusions to a more general case: particles in ultracold atom–multi-atom molecule conversion approximated as BEC systems trapped in asymmetric double wells (R≠0). To conduct further research, we introduced a new numerical calculation model. Based on the model in Section 2, we performed Schrödinger orthogonalization between instantaneous eigenstates $|E_{+}\rangle$ and $|E_{-}\rangle$, and defined this calculation process as a standard orthogonal transformation [45,46].

When *R* is not equal to zero, the affine-eigenstate transformation cannot be applied to the original system. Therefore, by using a standard orthogonal transformation, we can obtain solutions in more complex spatial environments. There are two reasons why we did not directly use a standard orthogonal transformation from the beginning: First, by comparing computational quantities, using an affine-eigenstate transformation calculation is simpler when *R* equals zero. Second, under an affine transformation, we can provide a numerical solution method, as shown in Section 3.

Definition:

(53) $$c_{1}=-NV{\left|a\right|}^{N-1}$$

(54) $$c_{2}=\frac{N+1}{2}R-\frac{1}{2}\sqrt{{\left(N+1\right)}^{2}R^{2}+4NV^{2}{\left|a\right|}^{2\left(N-1\right)}}$$

For the detailed proof, see the Appendix A. We can readily obtain:

(55) $$\left\{\begin{array}{cc} \dot{\theta } & =\left(H_{22}-H_{11}\right)+\frac{\left(H_{12}+H_{21}\right)\;s+\left(H_{21}-H_{12}\right)}{\sqrt{1-s^{2}}}\;\cos\theta ,\\ \dot{z} & =\left(H_{12}+H_{21}\right)\;\sqrt{1-s^{2}}\;\sin\theta . \end{array}\right.$$

**Figure 7:**
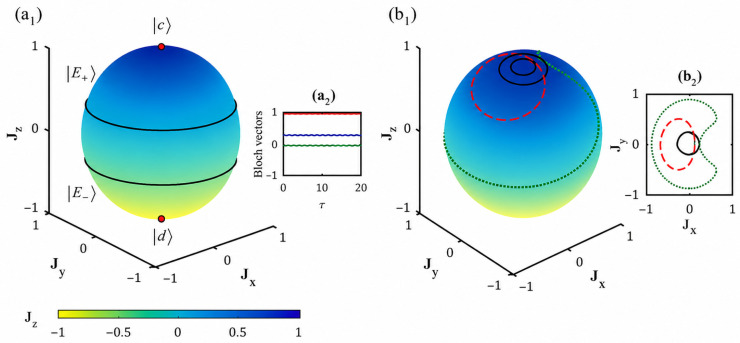
Figure (**a_1_**,**a_2_**) shows the instantaneous eigenstates under the standard-orthogonal transformation in self-trapping cases, where the red line represents the trajectory mapped on the z-axis of the Bloch sphere during self-trapping, the blue line represents the y-axis, and the green line represents the x-axis. Figure (**b_1_**,**b_2_**) shows the self-trapping plane trajectories when R≠0 while the other conditions remain unchanged, where the red line represents the trajectory change at R=2, the green line represents the trajectory change at R=1, and the black line represents the self-trapping plane at R=0 (with different initial conditions).

Based on the standard-eigenstate transformation, as shown in Figure 7b, self-trapping states are only related to whether *R* equals zero, that is, whether there exists an energy difference in the double well. When R≠0, the system originally in a self-trapping state will escape from the self-trapping plane; when the energy difference approaches infinity, the evolution trajectory becomes a point, as an infinitely deep well cannot be tunneled through.

By comparing the affine-eigenstate transformation with the standard-eigenstate transformation, we can find that these two transformations have a one-to-one correspondence, because Schmidt orthogonalization is a one-to-one transformation. Based on this, the conclusions obtained under these two different eigenstate transformations are universal.

Through eigenstate transformations (including affine and standard orthogonal transformations), we mapped the original system to a new representation space. This transformation introduces a new evolution parameter τ. Here, τ is not the laboratory time *t* and should not be interpreted as a new physical clock. It is an effective parameter used to organize trajectories in the transformed representation. At most, this role is heuristically reminiscent of the auxiliary time parameter used in geometric flows such as the Ricci flow equation

(56) $$\frac{\partial g_{ij}}{\partial t}=-2R_{ij}$$

where $g_{ij}$ is the metric tensor of the manifold and $R_{ij}$ is the Ricci tensor [47]. The analogy is limited to the use of an auxiliary deformation parameter; we did not construct a metric tensor for the physical state space, compute the curvature, or solve a Ricci-flow equation. The actual results of this paper come from the transformed mean-field equations, fixed-point conditions, and Jacobian analysis.

With dissipation, the unnormalized Bloch vector may move toward the origin because the total particle number decreases. The normalized population imbalance

(57) $$S=\frac{{\left|a\right|}^{2}-N{\left|b\right|}^{2}}{{\left|a\right|}^{2}+N{\left|b\right|}^{2}}$$

is used only to project the dissipative trajectory back to a normalized phase-space representation. This normalization is visually reminiscent of the volume-normalization term in normalized Ricci flow,

(58) $$\frac{\partial g_{ij}}{\partial t}=-2R_{ij}+\frac{2}{n}Rg_{ij}$$

where *n* is the manifold dimension and $\bar{R}=\frac{\int Rdv}{\int dv}$ is the average scalar curvature. However, Equation above is included only as an analogy for normalization under an auxiliary flow. We do not claim that the atom–molecule dynamics are governed by Ricci flow, nor did we use the Ricci curvature to derive the stability. The conservative statement is that the affine/orthogonal transformations provide a convenient geometric visualization: a non-canonical phase portrait can be represented on a standard Bloch sphere, where fixed points and their loss-induced stability changes are easier to inspect.

## 5. Phase-Modulated Control via Time-Dependent Magnetic Fields

In the previous two chapters, we assigned a constant value to the phase $\theta_{a}$, meaning that the initial phase $\theta_{a}$ would not change with time, remaining constant. In this chapter, we apply a time-varying phase magnetic field to the entire system, which affects the variation in $\theta_{a}$. We investigated the physical properties by considering $\theta_{a}$ and its numerical opposite $-\theta_{a}$ as a set of adjustable parameters.

We controlled the rate of phase change by adjusting the power of time, using the model:

(59) $$\theta_{a}=\omega_{a}\;\tau^{T}$$

where $\theta_{a}$ is our controlled phase, *t* is the time in the original system, and *T* is the power that affects the rate of τ variation.

According to the model, the phase and time are positively correlated, meaning that faster time changes lead to faster θ changes, and vice versa. Using the derivative of θ as the angular velocity, we deduced θ after applying the time-varying phase magnetic field by controlling the time-dependent changes in the angular velocity, and based on this, we analyzed how changes in θ affect the evolution trajectory.

Through a numerical simulation, we discovered that, when *T* takes values of 0, 1, and 2, four types of evolution trajectories with distinctly different properties emerge. Particularly when *T* equals 1, the evolution trajectories can be classified into two categories based on whether they cross the self-trapping plane.

(i) Consider classification based on whether the evolution trajectory crosses the self-trapping plane. When T>1, the evolution trajectories lay on both sides of the self-trapping plane; when θ>0, the trajectory was above the self-trapping plane, forming antisymmetric patterns with the evolution trajectory of −θ on the three-dimensional sphere. When T=1, by solving the first and second derivatives of θ, we found that both the angular velocity and the angular acceleration remained constant under this condition, and the evolution trajectory oscillated above the self-trapping plane without crossing it. When T<1, the evolution trajectory crossed the self-trapping plane while maintaining antisymmetric properties, with opposite evolution directions—evolving clockwise when θ>0 and counterclockwise when θ<0.

(ii) Consider classification based on the shape of the evolution trajectories. Focusing on cases where θ>0, when the power is greater than or equal to 2, the evolution trajectory shows a trend of spiraling upward toward the pole point on the sphere surface, without intersecting itself. When the power is between 1 and 2, the trajectory shows a zigzag pattern ascending to the pole point, with intersections occurring during ascent due to relatively slower conversion rates compared to when the power ≥2. When T=1, the evolution trajectory oscillated on one side of the self-trapping plane with a constant amplitude and period. When 0<T<1, the trajectory exhibited spiral oscillations near the self-trapping plane with an essentially constant amplitude. When T<0, the trajectory showed quasi-harmonic oscillations near the self-trapping plane, with an increasing amplitude and period. When T=0, a special case arose where the evolution trajectory’s extremity depended on the coefficient magnitude: when the coefficient before τ was between 0 and 2.22, the trajectory followed one cycle: self-trapping–clockwise oscillation on self-trapping plane–perpendicular to self-trapping plane–counterclockwise oscillation on self-trapping plane–self-trapping. When comparing positive and negative θ pairs, their evolution trajectories consistently followed antisymmetric principles.

**Figure 8:**
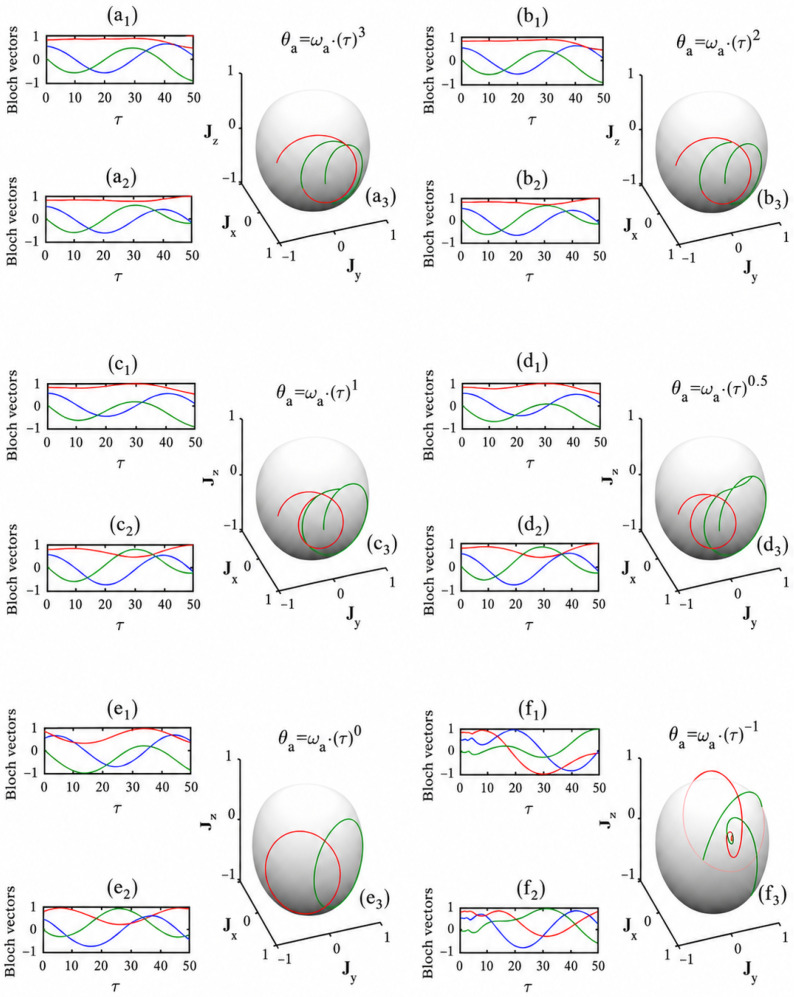
Phase-modulated control via time-dependent magnetic fields for six time-exponent protocols in the phase law $\theta_{a}\left(\tau \right)=\omega_{a}\tau^{T}$: (**a**) T=3, (**b**) T=2, (**c**) T=1, (**d**) T=0.5, (**e**) T=0, and (**f**) T=−1. In each case, panels with subscripts 1 and 2 show the time evolution of the Bloch-vector components as functions of the normalized time τ, and the panel with subscript 3 shows the corresponding trajectory on the Bloch sphere. In the time-evolution panels, the red, green, and blue curves denote $J_{x}$, $J_{y}$, and $J_{z}$, respectively. In the Bloch-sphere panels, red and green trajectories correspond to $+\theta_{a}$ and $-\theta_{a}$, respectively, with opposite rotation directions.

Based on the above situations, by controlling time to adjust the θ variations, different scenarios provide theoretical foundations for meeting different requirements in quantum control domains, as summarized in Figure 8.

## 6. Conclusions

In this work, we developed a phase-space framework for dissipative atom–molecule conversion dynamics and used it to clarify how self-trapping survives (or fails) in open environments. Starting from a Markovian master-equation description, we derived the corresponding mean-field equations and introduced an affine-eigenstate transformation that maps the original dynamical variables onto a standard Bloch-sphere representation. This mapping resolves the non-canonical geometric structure of the original variables and enables a unified identification of fixed points, trajectories, and stability in a transparent geometric picture. Focusing on the symmetric setting R=0, we showed that dissipation can convert fixed points into attractors or repellers, thereby inducing dissipative bifurcations, consistent with the broader understanding of loss- and noise-driven mean-field flows in bosonic systems. Crucially, our linear-stability analysis yielded a compact operational criterion, which controls whether the relevant fixed point becomes attractive or repulsive, which explains the strong sensitivity of ordinary self-trapping to the relative loss rates.

Beyond this conventional regime, we identified strict self-trapping as a critically balanced self-trapped state associated with S=0, for which the dissipative bifurcation mechanism becomes inactive. Consequently, the corresponding fixed point does not split into an attractor/repeller pair and the resulting localization remains exceptionally robust even for strong dissipation. We also distinguished pseudo self-trapping as a localized-like evolution that appears robust in time traces, but is not governed by a genuine self-trapped fixed point in the phase portrait.

Finally, by exploiting the one-to-one correspondence between steady solutions in the transformed representation and initial conditions in the original variables, we provided an inverse reconstruction route that can assist stability diagnoses and state preparation, and we discussed how time-dependent phase magnetic-field modulation can be used to steer trajectories for controlled manipulation. Future work may go beyond the mean-field description to quantify finite-particle-number corrections and benchmark the strict self-trapping regime in fully quantum simulations, for example, using quantum-trajectory techniques, and to explore experimentally relevant protocols for decoherence-robust state control.

Several limitations should be kept in view. First, the present theory is formulated at the mean-field level, so it captures mode amplitudes and phase-space stability, but not the full many-body entanglement spectrum. Second, the loss model is Markovian and contains single-particle atom and molecule loss; non-Markovian reservoirs, heating, technical phase noise, and three-body recombination can modify the stability margin. Third, the affine mapping is used only in the parameter regime where its determinant remains nonzero and where the conjugate condition justifies the Hamiltonian phase-space picture. These limitations define clear next steps: exact diagonalization or matrix-product simulations for finite particle numbers, quantum-trajectory calculations of mixed-state entropy and mode entanglement, and optimal-control protocols for preparing the S=0 strict self-trapping state. From the perspective of quantum information, the main implication is not that the present mean-field state is already a protected qubit, but that the strict self-trapping condition identifies a decoherence-insensitive operating point that could be useful for molecular qubit storage, state-transfer stabilization, and analog quantum-simulation protocols in noisy atom–molecule platforms.

## Data Availability

The data presented in this study are available from the corresponding author upon reasonable request.

## Appendix A. Stepwise Derivation of the Main Working Equations

This appendix collects the main algebraic steps used in the revised manuscript. First, applying the Lindblad master equation to the field operators gives

(A1) $$\frac{d\langle \hat{a}\rangle }{dt}=-i\langle \left[\hat{a},\hat{H}\right]\rangle -\frac{\Gamma_{a}}{2}\langle \hat{a}\rangle ,\;\frac{d\langle \hat{b}\rangle }{dt}=-i\langle \left[\hat{b},\hat{H}\right]\rangle -\frac{\Gamma_{b}}{2}\langle \hat{b}\rangle .$$

Using $\left[\hat{a},{\hat{a}}^{†}\hat{a}\right]=\hat{a}$, $\left[\hat{a},{\left({\hat{a}}^{†}\right)}^{N}\right]=N{\left({\hat{a}}^{†}\right)}^{N-1}$, and the corresponding relation for $\hat{b}$, and then replacing operator moments by coherent amplitudes, yields Equation (4). A common scalar phase may be subtracted from both amplitude equations without changing the normalized phase portrait. With

(A2) $$R=\frac{1}{2N}\left(N\mu_{a}-\mu_{b}+NU_{aa}-\frac{1}{N}U_{bb}\right),\;U=\frac{1}{2N}U_{ab}-\frac{1}{2}U_{aa}-\frac{1}{2N^{2}}U_{bb},\;s={\left|a\right|}^{2}-N{\left|b\right|}^{2},$$

the remaining relative detuning and nonlinear interaction terms reduce to the diagonal entries of Equation (6), while the conversion commutators give the off-diagonal factors $NV{\left(a^{*}\right)}^{N-1}$ and $Va^{N-1}$.

Second, the affine mapping used in Equations (11)–(15) also gives the population-imbalance formula used in Equation (16). Let $\phi =\left(N-1\right)\theta_{a}$, $D=\sqrt{{\left(N+1\right)}^{2}R^{2}+4N\nu_{0}}$, and $A_{N}=NV{\left|a_{0}\right|}^{N-1}$. Then,

(A3) $$a=-A_{N}\left(e^{-i\phi }c+d\right),\;b=E_{R+}c+E_{R-}e^{i\phi }d.$$

With $s_{1}={\left|c\right|}^{2}-{\left|d\right|}^{2}$, $s_{2}={\left|c\right|}^{2}+{\left|d\right|}^{2}$, and $\zeta =c^{*}de^{i\phi }+cd^{*}e^{-i\phi }$, direct expansion gives

(A4) $$\begin{array}{cc} {\left|a\right|}^{2} & =A_{N}^{2}\left(s_{2}+\zeta \right),\\ {\left|b\right|}^{2} & =\frac{E_{R+}^{2}+E_{R-}^{2}}{2}s_{2}+\frac{E_{R+}^{2}-E_{R-}^{2}}{2}s_{1}+E_{R+}E_{R-}\zeta . \end{array}$$

Using $E_{R\pm }=\frac{N+1}{2}R\mp \frac{1}{2}D$ and $A_{N}^{2}=N^{2}\nu_{0}$, the original imbalance becomes

(A5) $$\begin{array}{cc} s={\left|a\right|}^{2}-N{\left|b\right|}^{2}= & \frac{N(N+1)}{2}RD\;s_{1}-\frac{N{\left(N+1\right)}^{2}}{2}R^{2}\;s_{2}+2N^{2}\nu_{0}\;\zeta , \end{array}$$

which is Equation (16). This derivation also fixes the coefficient in the first row of M(t) as $-A_{N}=-NV{\left|a_{0}\right|}^{N-1}$, consistent with the eigenvectors in Equations (8) and (9).

Third, for any nonsingular instantaneous-eigenstate matrix M(t), the transformed amplitudes obey

(A6) $$i\frac{d}{d\tau }\psi_{cd}=\left(M^{-1}H_{t}M-iM^{-1}\dot{M}\right)\psi_{cd}.$$

The four entries of $M^{-1}H_{t}M$ produce the dynamical terms in Equation (25), and $-iM^{-1}\dot{M}$ gives the Berry-connection matrix elements $E_{++}$, $E_{+-}$, $E_{-+}$, and $E_{--}$. This is the origin of the geometric couplings used in the transformed Hamiltonian.

Fourth, the fixed points are obtained by solving $\dot{s}=0$ and $\dot{\theta }=0$. Linearizing around each solution $\left(s_{0},\theta_{0}\right)$ gives

(A7) $$\left(\begin{array}{c} \delta \dot{s}\\ \delta \dot{\theta } \end{array}\right)=J\left(s_{0},\theta_{0}\right)\left(\begin{array}{c} \delta s\\ \delta \theta \end{array}\right)+O\left(\delta^{2}\right),\;\lambda_{\pm }=\frac{\mathrm{Tr}\;J\pm \sqrt{{\left(\mathrm{Tr}\;J\right)}^{2}-4\det J}}{2}.$$

For the closed Hamiltonian flow satisfying the conjugate condition, TrJ=0, so purely imaginary eigenvalues identify center-type stable fixed points and real eigenvalues identify saddle-type unstable points. For the open system, the loss term modifies the trace according to Equation (47), producing the attractor/repeller sign criterion used in Figure 5.
